# Clustering Infertile Couples With Dyadic Approach: WHO-5-WBI as a Promising Tool for Assessing Psychological State

**DOI:** 10.5334/pb.539

**Published:** 2020-06-18

**Authors:** Borbála Eszter Hegyi, Krisztián Kocsis, András Király, Csaba Kazinczi, Bálint Ando, Ildikó Kovács, Zoltán Kozinszky, Gábor Németh, Norbert Pásztor

**Affiliations:** 1 Department of Obstetrics and Gynecology, University of Szeged, Szeged, HU; 2 Department of Neurology, University of Szeged, Szeged, HU; 3 Department of Psychiatry, University of Szeged, Szeged, HU; 4 Department of Obstetrics and Gynaecology, Danderyd Hospital, Stockholm, SE

**Keywords:** Infertility, depression, anxiety, dyadic approach, WHO-5 Well-Being Index

## Abstract

Infertility may be associated with severe psychological burden and many couples need mental support. We used dyadic approach to identify couples with disturbed psychological condition and we tested the WHO-5 Well-Being Index (WHO-5-WBI) questionnaire as a possible, rapid screening method. Extensive psychological assessment of infertile couples was carried out with Beck’s Depression Inventory, Spielberger State-Trait Anxiety Inventory, WHO-5-WBI, Symptom Check List-90 Revised Test, Fagerstrom Test for Nicotine Dependence, Alcohol Use Disorders Identification Test. Data of 128 patients (64 couples) were used in the statistical calculations. The Two-Step cluster analysis has revealed 2 groups, which could be separated supremely based on the level of experienced depression, anxiety and according to the general mental health. The WHO-5-WBI questionnaire showed consistent results while classifying couples into groups, which were formed. Our results indicate that infertility affects both spouses almost in the same extent in several psychological aspects. A cluster of couples with increased psychological burden could be clearly separated. The WHO-5-WBI questionnaire was a promising tool to screen reliably spouses based on their psychological state and identify couples that need psychological support during their fertility work-up and treatment.

## Introduction

Infertility can be described as an inability to become conceived within one year despite regular sexual intercourse without contraceptive usage (Jayashankar, 2017). Approximately 186 million people are facing the problem of infertility worldwide and in Hungary 9 percentage of the population are affected (Vander Borght & Wyns, 2018). Even though this phenomenon has been remedied for decades with advanced assisted reproduction techniques, involuntary childlessness and its psychological consequences are mostly neglected and barely emphasized reproductive healthcare problems (Whittaker, Inhorn, & Shenfield, 2019).

Infertility causes a ponderous psychological burden both for women and men, including depression, anxiety, feeling shame and low self-esteem, which negatively influence the coping mechanism of individuals and are likely to reduce the success of the future fertility treatment (Baqutayan, 2015; Malina, Blaszkiewicz, & Owczarz, 2016; McQueeney, Stanton, & Sigmon, 1997; Taebi, Simbar, & Abdolahian, 2018). Younglai and colleagues also highlight the observation that depression and anxiety in women and stress in men might be amongst the main causes of fertility problems and can also negatively affect the process of *in vitro* fertilization (IVF) programs. Therefore, it would be worthwhile to pay particular attention to the assessment of the psychological state of those who are involved in the fertility programs (Younglai, Holloway, & Foster, 2005).

In the literature, only a few publications can be found about the infertility related psychological interdependence between spouses, which is called dyadic approach. Describing as a whole system, the interpretation of dyadic approach can be an important tool to understand the characteristics of involuntary childlessness, since in an intimate relationship the spouses may affect each other’s mental state reciprocally (Peterson, Pirritano, Christensen, & Schmidt, 2008).

In this study, a model free clustering approach was used to determine the main psychological aspects allowing to differentiate infertile couples based on their experienced level of depression, anxiety and general mental health, as primary outcome measures. Using numerous screening instruments, we intended to identify those couples who are facing infertility related psychological distress, thus can benefit from further psychological counseling. Considering the general experiences, that family doctors and hospital care staff are facing lack of resources and time, a focused screening for psychological disturbances of infertile patients would be useful. As a short, quickly applicable instrument, the WHO-5 Well-Being Index (WHO-5-WBI) was a promising tool for assessing psychological parameters of patients in a previous review (Topp, Ostergaard, Sondergaard, & Bech, 2015). We hypothesized, that WHO-5-WBI could substitute extensive psychological examination due to its reliability and specificity (Topp et al, 2015). To prove our hypothesis, we applied the WHO-5-WBI on our study population and compared the results to the scores of the other tests to determine whether WHO-5-WBI could be used in a daily clinical practice to assess the psychological state of the infertile couples.

## Materials and Methods

### Design

We managed a cross-sectional research design with a dyadic approach to examine the interdependence of the male and female spouses’ psychological state related to infertility. We used the Hungarian version of psychometrical instruments to measure depression (Beck’s Depression Inventory – BDI), anxiety (Spielberger State-Trait Anxiety Inventory – STAI), well-being (WHO-5 Well-Being Index – WHO-5 WBI), general mental state (Symptom Check List – 90 Revised Test – SCL-90R), nicotine dependence (Fagerstrom Test for Nicotine Dependence – FTND), alcohol dependence (Alcohol Use Disorders Identification Test – AUDIT). Our primary outcome measures were the values of the level of depression, anxiety, general mental-health and well-being index. Accordingly, each case as one couple had two test results on each instrument. We identified the main characteristics of the formed clusters and examined whether the responses to the WHO-5 WBI fit the spouses into the formed groups. In addition, the data of WHO-5 WBI, as an independent variable, were not used in the calculation process of clustering.

### Patients

Infertile couples who attended to infertility examination were enrolled in this study at an Andrology Outpatient Clinic during a time period from August 2017 to April 2019. The study was approved by the Regional Ethical Committee (No.: 196/2017-SZTE). All subjects received a written informed consent in accordance with the Declaration of Helsinki. The main exclusion criteria were the presence of any previous psychiatric disorder and/or any severe case in life story.

### Instruments

#### Primary Outcome Measures

##### Hungarian version of Shortened Beck’s Depression Inventory (BDI)

Nine-item shortened version of Beck Depression Inventory with 21 questions, a four-point (from 0 to 3) Likert scale questionnaire asks for symptoms of depression such as social withdrawal, indecision, sleep disturbance, fatigue, excessive anxiety due to physical symptoms, disability, pessimism, satisfaction and lack of joy and self-blame. The total score is 63 points, normal scores of the test differ from 0 to 9, scores above this range indicate more depressive symptoms (Beck, Ward, Mendelson, Mock, & Erbaugh, 1961). BDI served as a dependent variable of cluster analysis.

##### Hungarian version of State-Trait Anxiety Inventory Form Y-1 and Form Y-2 (STAI)

The State-Trait Anxiety Inventory being widely used in clinical practice and research. It is a short self-report questionnaire to evaluate the level of anxiety and was designed by Spielberger and colleagues. Form STAI-State and STAI-Trait measure state and trait anxiety with 20-20 items (Spielberger, Gorsuch, Lushene, Vagg, & Jacobs, 1983). In Hungary, the STAI-State has a normal value for women at 42,6 (SD ± 10,79) points, and normal value for men at 38,4 points (SD ± 10,66). In the STAI-Trait questionnaire, the normal value for women is 45,3 (SD ± 7,97) point and the normal value for men is 40,9 (SD ± 7,78) points (Sipos, Sipos, & Spielberger, 1994). Higher scores refer to higher level of anxiety. Some authors make allowance for STAI as a measurement tool of general negative affect, for instance anxiety, depression and well-being. STAI-State and STAI-Trait served as dependent variables of cluster analysis.

##### Hungarian version of Symptom Checklist-90 Revised test (SCL-90R)

The SCL-90R is a multidimensional self-reported questionnaire to assess nine different dimensions: somatization, obsessive–compulsive, interpersonal sensitivity, depression, anxiety, hostility, phobic anxiety, paranoid ideation, and psychotic symptoms. Each dimension can be interpreted separately. Total scores can range from 0 to 450, higher scores in SCL-90R refer to worse general psychological state (Derogatis, Lipman, Rickels, Uhlenhuth, & Covi, 1974). To determine general mental health, we calculate the Global Severity Index (GSI) (Total Score/90), which value is 0,62 (SD ± 0,50) in the validated normal Hungarian sample. SCL-90R served as a dependent variable of cluster analysis.

##### Hungarian version of WHO-5 Well-Being Index (WHO-5-WBI)

The World Health Organization-Five Well-Being Index is a short self-reported questionnaire containing five items to evaluate current mental well-being in a time period two weeks prior the completion. The questionnaire is based on the WHO-10 Well-Being Index and was developed by Bech in collaboration with the World Health Organization. The instrument contains five different questions about subjective psychological well-being as daily activity; being vigorous; being cheerful; being calm, relaxed; and general interest in life. Each item is scored from 0 to 3, the maximum possible score is 15 (Heun, Burkart, Maier, & Bech, 1999). The average score of men is 8,2 (SD ± 2,7), for women is 7,4 (SD ± 3,8), measured on a validated Hungarian sample. Higher WHO-5-WBI scores mean better well-being of the subjects. The test was used as independent variable while assessing its diagnostic efficacy.

#### Additional instruments

##### Hungarian version of Alcohol Use Disorders Identification Test (AUDIT)

AUDIT is a 10-item short self-reported questionnaire to examine the frequency of alcohol consumption (three questions), the rate of alcohol dependence (three questions) and the derived problems due to the alcohol consumption as well (four questions). Each question is scored from 0 to 4, the maximum possible score is 40. AUDIT provides a simple method of early detection of hazardous and harmful alcohol use in primary health care. Higher scores (above 8 points) in AUDIT refer to more severe alcohol dependence (Saunders, Aasland, Babor, de la Fuente, & Grant, 1993).

##### Hungarian version of Fagerstrom Test for Nicotine Dependence (FTND)

The FTND is a six-item short self-reported questionnaire to assess the rate of physical addiction to nicotine. The inventory briefly covers smoking habits, such as using a first cigarette a day, smoking control or smoking during illness. The possible maximum score is 10, from 0 to 2 points there is no sign of nicotine dependence. Higher Fagerstrom scores refer to more intense addiction to nicotine (Fagerstrom, 1978).

### Statistical analyses

The data of each couple, both male and female were ordered into the same case. Considering the fact, that the scores of WHO-5-WBI for males and WHO-5-WBI for females are independent variables in the classification process, we examined them first for normality and outliers. We experienced one outlier on the scale of WHO-5-WBI among females. Avoiding the distortion, we excluded this case (couple) from the analysis. Health related additional instruments, Fagerstrom and AUDIT results were analyzed as basic characteristic variables.

We found that WHO-5-WBI scores differed from normal distribution for both genders, therefore Logistic Regression was used in the later statistical analysis to evaluate its predictive effectiveness.

To determine the interdependence between the male and female partner’s psychological involvement (e.g.: depression, anxiety, general mental health), we separated the couples into two clusters, based on BDI-male, BDI-female, STAI-State-male, STAI-State-female, STAI-Trait-female, SCL-90R-male, SCL-90R-female results. Two-Step cluster analysis was carried out consecutively, because it considered as a robust method against a lack of normal distribution and outliers. We also determined the main characteristics of the formed groups.

As previously defined, the reliability of WHO-5-WBI classification was tested with Logistic Regression on the clusters. Reaffirming the diagnostic values of WHO-5-WBI, we performed ROC-analysis and determined the effectiveness of WHO-5-WBI.

Data were analyzed using the Statistical Package for Social Sciences (SPSS 25.0.0 for Windows, SPSS Inc., http://www.spss.com).

## Results

### Sample characteristics

All the questionnaires were self-completed and 61% of the infertile couples agreed to participate in the study and complete the questionnaires. Altogether, 65 infertile couples were enrolled. Later, we excluded one outlier couple during the statistical data analysis. Accordingly, the final calculations were performed with 64 case (n = 128).

The results of the questionnaires in our study population are shown in Table 1. The average age of men was 37.34 years (±5.84 SD); of women 34.07 years (±0.06 SD). In terms of education, most common highest level of education was high school degree for both genders (32.8% of men; 37.5% of women). Mean BDI results were 3.59 for males and 5.17 for females. Mean scores of STAI-Trait and STAI-State tests were 33.61 and 34.73 for men, 37.36 and 37.31 for women. Mean SCL-90R-GSI was 0.33 for both genders, WHO-5-WBI mean scores was similar, 9.59 for men and 9.56 for women. With regard to smoking (FNTD) and alcohol consumption (AUDIT), 68.8% of men were non-smoker, 28.3% were moderate smoker, the rest of them (2.9%) reported severe nicotine dependency. Regarding the women, 75.0% were non-smoker, 25.0% were moderate smoker and no serious nicotine addict was registered. Among men in terms of alcohol consumption, 12.5% were non-drinker, 71.9% were moderate drinker, the remainders (15.6%) were facing serious alcohol problems. In parallel, 25% of women do not consume alcohol, 68.7% can be identified as moderate drinker, the remainders (6.3%) having serious alcohol problems.

**Table 1:** Descriptive statistics showing the main characteristics of domains for mental health between males and females, with test of normality.

	Mean (± SD)			Differences between male and female		Test of Normality (Saphiro-Wilk) *p* value	

	Couples	Male	Female	t (df = 63)	p	Male	Female

n	64	64	64				
Age	35.71 (0.59)	37.35 (0.73)	34.08 (0.60)	5.129	<0.001*	0.655	0.248
FTND	1.27 (0.24)	1.42 (0.29)	1.10 (0.25)	1.186	0.240	<0.001	<0.001*
AUDIT	3.10 (0.38)	3.96 (0.49)	2.25 (0.34)	4.338	<0.001*	<0.001	<0.001*
BDI	4.38 (0.50)	3.59 (0.59)	5.17 (0.66)	–2.110	0.039*	<0.001	<0.001*
STAI-State	36.02 (1.02)	34.73 (1.20)	37.31 (1.22)	–1.966	0.054	0.008	0.327
STAI-Trait	35.48 (0.91)	33.60 (0.94)	37.35 (1.13)	–3.759	<0.001*	0.196	0.060
SCL-90-R (GSI)	0.33 (0.25)	0.28 (0.29)	0.37 (0.29)	–2.392	0.020*	<0.001	<0.001*
WHO-5-WBI	9.57 (0.30)	9.59 (0.40)	9.56 (0.29)	0.090	0.928	0.055	0.031*

*Notes*: BDI: Beck’s Depression Inventory; STAI: State-Trait Anxiety Inventory; SCL-90-R: Symptom Checklist-90-Revised test; WHO-5-WBI: WHO-5 Well-Being Index; FTND: Fagerstrom Test for Nicotine Dependence; AUDIT: Alcohol Use Disorders Identification Test. Significant *‘p values’* are signed with *.

Two-step Cluster Analysis generated two distinct cluster groups with highly homogenous patterns of health-related psychological characteristics. Of the 64 couples, 53.1 % (n = 34) can be classified as Cluster 1: “Infertile couples with high values on mental health inventories” and 46.9 % (n = 30) as Cluster 2: “Infertile couples with low values on mental health inventories”.

### Cluster profiles

Cluster 1 produced higher levels on health-related and psychologically relevant questionnaires, in contrast, the couples in Cluster 2 showed lower results. According to the cluster analysis process, all variables showed a significant difference between the two clusters. In the Cluster 1, for both men and women higher average levels of anxiety were experienced on STAI-Trait (STAI-Trait Cluster 1: men = 38.32; women = 43.03; Cluster 2: men = 28.27; women = 30.93). STAI-State also showed elevated scores by each gender in Cluster 1 (STAI-State Cluster 1: men = 40.44; women = 43.00; Cluster 2: men = 28.27; women = 30.87). BDI results also suggested that members of the Cluster 1 experience inferior conditions compared to the Cluster 2 group (BDI scores, Cluster 1: men = 5.97; women = 7.94; Cluster 2: men = 0.90; women = 2.03). Specifying the mental-health condition in general (measured with SCL-90R), we experienced higher total scores for each gender (GSI scores: men = 0.45; women = 0.55) in Cluster 1, compared to Cluster 2 (men = 0.10; women = 0.18). In addition, men in Cluster 1 displayed higher risk for alcohol dependency (AUDIT Cluster 1: t(62) = 49.505, *p* = 0.021). The age and the level of nicotine addiction showed no significant difference between the clusters. The results are summarized in Table 2.

**Table 2:** The main features of calculated clusters showing significant differences on mental health scales estimating higher or lower theoretical probability of occurring mental health problems.

	Mean Scores						

	Cluster 1 Infertile couples with higher-risk of mental health issues			Cluster 2 Infertile couples with lower-risk of mental health issues			Between cluster differences (*p*-value)

	Male	Female	*p*-value	Male	Female	*p*-value	

n	34	34		30	30		
STAI-Trait	38.32	43.03	0.958	28.27	30.93	0.481	<.001*
STAI-State	40.44	43.00	0.287	28.27	30.87	0.371	<.001*
SCL-90R (GSI)	0.45	0.55	0.331	0.10	0.18	0.247	<.001*
BDI	5.97	7.94	0.624	0.90	2.03	<.001*	<.001*

*Notes*: Within cluster comparison suggests interdependence between male and female partners, namely we found no significant differences, only in the case of BDI within Cluster 2. Significant ‘*p* values’ are signed with *. BDI: Beck’s Depression Inventory; STAI: State-Trait Anxiety Inventory; SCL-90R: Symptom Checklist-90-Revised test; GSI: Global Severity Index.

### Predictive efficiency of WHO-5-WBI

Logistic regression analysis was performed to assess the predictive efficiency of WHO-5-WBI on the likelihood that the infertile couples would be classified into the Cluster 1 or Cluster 2. Regarding to this, we used the cluster membership as the dependent variable in the logistic regression. The model representing predictors was statistically significant (χ2 (df 2, n_total_: 64, n_cluster1_: 26, n_cluster:_ 38) = 14.59, *p* < 0.0001), explaining that the model was able to distinguish between infertile couple who were separated into clusters based on their results of BDI, STAI, SCL-90R (see Table 3). The results also show that WHO-5-WBI-male and WHO-5-WBI-female, as independent predictor variables, specify the regression with a significantly negative coefficient (WHO-5-WBI-male: 0.298, *p* = 0.016; WHO-5-WBI-female: 0.474, *p* = 0.008). Congruent association of predicted probabilities and observed responses was 75.0%, which is further evidence of the effectiveness of the classification. Despite the fact that couples were interpreted as cases (dyads), the values of both women and men, had a reliable diagnostic model for the couple’s mental state.

**Table 3:** Results show significant estimator effect for both variables (WHO-5-WBI-male and WHO-5-WBI-female).

Estimator effect	B	df	Wald χ2	Pr > χ2	

WHO-5-WBI-male	–0.298	1	5.837	0.016*
WHO-5-WBI-female	–0.474	1	6.943	0.008*

*Notes*: Suggesting the efficacy of WHO-5-WBI (WHO-5 Well-Being Index) scale while classifying cases into the generated clusters. Based on this, we can conclude that WHO-5-WBI has a good predictive effect deciding later cluster membership. Significant *‘p values’* are signed with *.

**Figure 1:**
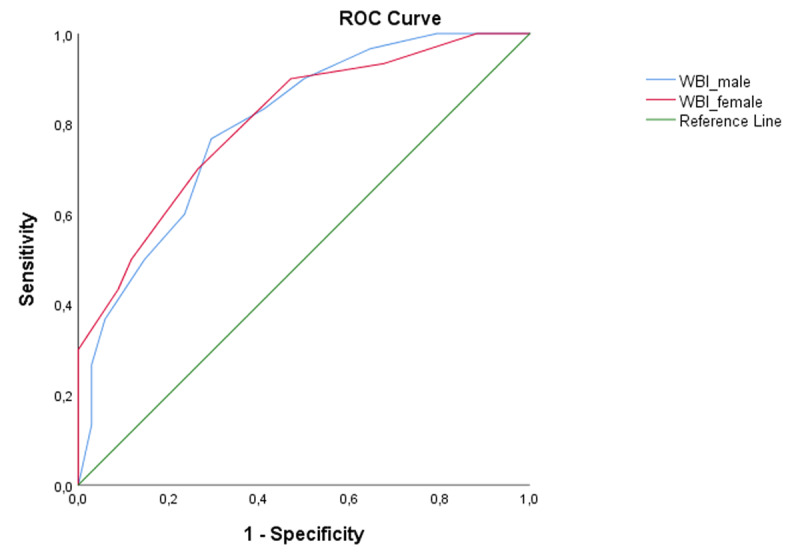
Result of the logistic regression analysis to assess the efficacy of WHO-5-WBI in classifying infertile couples. *Notes*: The logistic regression analysis showed that both the values of the WHO-5-WBI questionnaire of male and female spouses were reliable predictors for the infertile couple’s mental state (The curve of WBI_male (blue line), and WBI_female (red line) do approximate to higher sensitivity as well as to lower 1-specificity values). WHO-5-WBI: WHO-5 Well-being Index; ROC Curve: Receiver Operating Characteristic Curve; WBI_male: WHO-5-WBI values in male spouses; WBI_female: WHO-5-WBI values in female spouses.

For further confirmation, and showing the tradeoff between sensitivity and specificity, a Receiver Operating Characteristic curve was calculated. The appropriate ROC curve was drawn in (Figure 1) (AUC_WHO-5-WBI-male_ = 0.797, 95% confidence interval: 0.689-0.904, p < 0.001; AUC_WHO-5-WBI-female_ = 0.804, 95% confidence interval: 0.699-0.910, p < 0.001). The ROC analysis suggests that WHO-5-WBI as a diagnostic test has separative ability to discriminate between cluster memberships.

## Discussion

Infertility is a concern of public health and it may be presented with serious psychological consequences. Freeman and colleagues demonstrated that the involuntary childlessness affects both spouses, 50% of female and 15% of male spouses subsisted it as the most grievous life crisis (Freeman, Boxer, Rickels, Tureck, & Mastroianni, 1985). Volgsten and colleagues revealed that 10.9% of female and 5.1% of male spouses suffered from major depression due to infertility, and also the presence of anxiety was trenchant in both genders (Volgsten, Skoog Svanberg, Ekselius, Lundkvist, & Sundstrom Poromaa, 2008). According to previous studies, the spouses may influence the mental well-being of each other, so a dyadic approach was introduced in the psychological care (Kim, Shin, & Yun, 2018; Peterson et al, 2008). Couples with frail mental health need professional support to improve satisfaction and the outcome of the assisted reproductive treatments.

We aimed to identify couples at risk of inferior psychological condition. As an extended screening, several instruments (BDI, STAI, SCL-90R, WHO-5-WBI, AUDIT, FTND) were used in our study. A model free clustering approach was used, and the interdependence of spouses was analyzed regarding the infertility related psychological burden. The results revealed that infertile couples could be separated based on the scores of SCL-90R, STAI-State – Trait and the BDI, we were able to identify two, significantly different clusters, one (Cluster 1) with relatively high and other (Cluster 2) group with relatively low scores. Cluster 1 could be typified as one in which spouses experienced more expressed infertility related psychological symptoms, in contrast, couples in Cluster 2 presented lower level of anxiety and depression. Male and female psychological conditions were similar within the clusters, supporting the interdependence between the spouses. Furthermore, a trend was observed that women experienced higher levels of perceived stress and depression in general, compared to men in both clusters. Our results are in line with the literature, Kim and colleagues investigated how infertility related stress and depression affects the quality of life among spouses: they found that females showed higher depression scores compared to their male partner. On the other hand, they investigated that the level of depression and anxiety level of females affected directly the level of depression and anxiety level of male spouses, even though this interference was not reciprocal (Kim, Shin, & Yun, 2018). Similar results were reported form an Italian study. Women showed threefold incidence for anxiety and 2.5-fold incidence for depressive symptoms during *in vitro* fertilization process, compared to men. Furthermore, women with depressive and anxiety symptoms tended to have a partner with abnormal level of anxiety. In the same population, depressed and anxious men often had a spouse with problematic psychological status (Chiaffarino et al, 2011). Our data confirmed that the infertility related depression and anxiety were the primary symptom dimensions and global indices derived from SCL-90-R affected spouses with a similar impact.

Our results may promote significant changes in the psychological care of infertile patients. During the course of the fertility treatment, couples identified with increased anxiety or depression need mental support. Clustering patients can make the utilization of healthcare services more effective, since in our study population only 53.1% of the couples needed special support from experts, the remaining 46.9% of patients were in good mental condition. Based on the results, it is advisable to handle spouses as a unit during the psychological counseling rather than using individual approaches. However, such a complex testing used in our study is time consuming, may present inconvenience for the patients and the medical staff, and special knowledge is required for the interpretation. The access to professionally competent and adequately resourced staff is often limited during the assisted reproductive courses, a quick, accurate screening method for couples with inferior psychological state would be useful. For this purpose, we applied the WHO-5-WBI to our study population, whether this short, simple test is appropriate for distinguishing between the clusters, which were based on multiple instruments (BDI, STAI-S, STAI-T, SCL-90R). It might be used as a first line screening by general practitioners or IVF nurses, and couples with low scores can be referred to professionals for further psychological care. In a recent review, Topp and colleagues pointed out that the WHO-5-WBI is a promising tool for assessing psychological parameters of patients, including depression and anxiety (Topp et al, 2015). The main argument for using the WHO-5-WBI is that it contains only 5 items and can be applied quickly during the exploration phase (Heun et al, 1999). Other studies also support the use of the WHO-5-WBI to detect psychological parameters (Gariepy, Honkaniemi, & Quesnel-Vallee, 2016; Henkel et al, 2003), and despite cultural differences, the accuracy and reliability of the index show a relatively consistent result (Saxena, Carlson, Billington, & Orley, 2001). During the logistic regression analysis, WHO-5-WBI results as independent variables changed contrary to the values of the cluster variables. The higher level of the WHO-5-WBI predicts lower scores on the scales of BDI, STAI, SCL-90R. This result and ROC analysis support that WHO-5-WBI questionnaire may be a useful tool in short mental-health assessment, it had a good separative ability on general mental health. In our study sample, the WHO-5-WBI was suitable for determining the two clusters and identified couples with elevated level of psychological burden with good accuracy. When a couple is screened out, an expert has to decide regarding further diagnostic or therapeutic steps, and with this method, an increased diagnostic efficiency and more targeted care can be achieved. It has to be decided in further research, whether the WHO-5-WBI questionnaire can have a role in the follow-up process and it is suitable for monitoring the improvement in the psychological state of the counseled couples. Furthermore, it is also a subject of interest, what is the optimal repetition frequency of the psychological screening, because the mental condition of the couples can worsen during the prolonged fertility treatment, or in case of failed IVF cycles (Newton, Hearn, & Yuzpe, 1990).

We observed a relatively high incidence of severe alcohol consumption (15.6% for males and 6.3% among females) and only a low number of strongly nicotine-dependent patients (2.9% of males, none of females). In Hungary, 7–11% of men between 18–64 years of age are reported as seriously alcohol-dependent, and only about 1% of women (Központi Statisztikai Hivatal, 2015). It has to be a caution for the health care practitioners that the level of alcoholism in the infertile population exceeds the national average, not only in the view of the negative effect of alcohol on the reproductive health, but also counting the psychosocial consequences. It is notable, that distressed men (Cluster 1) are at elevated risk, particularly. The relatively low number of heavy smokers is satisfying, but 31.2% of men and 25% of women are still smoking during the course of fertility work-up and treatment. However, the percent of smokers is a slightly lower than in the general Hungarian population at the age of 18-64 (35-42% for men, 27-28% for women) (Központi Statisztikai Hivatal, 2015). The data on alcohol consumption and smoking underline the importance of patient education regarding the unhealthy habits and lifestyle factors.

## Limitations

Undoubtedly, our study has some limitations. The ratio of infertile couples that attend at infertility specialist in developed countries is around 45–56% (Boivin, Bunting, Collins, & Nygren, 2007), therefore, the generalization of our results has to be handled prudentially. Furthermore, only 61% of invited couples took part in the research, which may affect our conclusion. It is possible, that couples in the worst psychological conditions did not fill the questionnaires. Hopefully, using only a short test as the WHO-5-WBI may increase the willingness of couples to participate in the psychological screening. Another limitation of our study is that we have not included questionnaires examining the coping strategies of the spouses, though demonstrative data are available the relation between the level of infertility related stress and the different coping mechanisms (Peterson et al, 2008). Questionnaires for the coping strategies would have made the testing uncomfortably lengthy for the couples resulting a further decrease in the participation rate.

## Conclusion

As we know, this is the first study, which was able to classify couples into two significantly different clusters regarding the infertility-related psychological burden. The mental conditions of the spouses were interdependent and similar; they were assigned into the same cluster allowing us to handle them as a dyad. However, scores from BDI, STAI, and SCL-90R questionnaires characterized mostly the mental health of the couples, as a screening method, the short WHO-5-WBI test also was able to identify couples with significant psychological burden. These patients need professional mental support during the infertility treatment, and we believe, based on our results, that WHO-5-WBI is a convenient tool for health care providers and the patients to identify the couples at need.
